# Clinical features of ROHHAD syndrome and treatment outcomes following rituximab therapy: a descriptive case series of Chinese children

**DOI:** 10.3389/fped.2026.1960692

**Published:** 2026-10-26

**Authors:** Danyu Song, Xingzhi Chang, Yao Zhang, Ye Wu, Weihua Zhang, Jiuwei Li, Taoyun Ji

**Affiliations:** 1 Department of Pediatrics, Peking University First Hospital, Beijing, China; 2 Department of Neurology, Beijing Children’s Hospital, Capital Medical University, National Center for Children’s Health, Beijing, China; 3 Department of Pediatrics, PKUFH-NINGXIA Women & Children’s Hospital, Yinchuan, China

**Keywords:** clinical features, neuropsychiatric abnormalities, obesity, rituximab, ROHHAD syndrome

## Abstract

**Objective:**

We aimed to delineate the clinical phenotypes and symptom evolution of Rapid-onset Obesity with Hypothalamic dysfunction, Hypoventilation, and Autonomic Dysregulation (ROHHAD) syndrome in Chinese children and to evaluate the real-world efficacy of rituximab therapy.

**Methods:**

We retrospectively reviewed the medical records of seven children diagnosed with ROHHAD syndrome between 2010 and 2025. Clinical data on baseline characteristics, core symptoms, tumor status, and therapeutic responses were extracted and analyzed using descriptive statistics.

**Results:**

Among the 7 patients, there were 3 males and 4 females. The median age of onset was 2.8 years (2.0–7.9 years), and the median age at diagnosis was 3.8 years (3.5–8.5 years). The median diagnostic delay was 1.2 years (range, 0.5–3.6 years). All patients exhibited the triad of rapid-onset obesity, hypothalamic dysfunction, and autonomic dysregulation. The median ages of onset for obesity, hypothalamic dysfunction, and autonomic dysregulation were 3.2, 3.1, and 3.4 years, respectively. Neuropsychiatric abnormalities were universal (7/7, 100%), emerging concurrently with or shortly after weight gain. Central hypoventilation was observed in 5 patients (71.4%). Neural crest-derived tumors were found in 4 patients (57.1%). Cardiac involvement was present in 4 patients (57.1%), with 3 cases developing severe heart failure. All patients had neurological abnormalities, with 2 cases accompanied by epileptic seizures. Interleukin-6 (IL-6) levels were tested in 4 patients, with elevation in 2 cases. Six patients received first-line immunotherapy (corticosteroids/intravenous immunoglobulin) with poor response. Three patients received rituximab; all showed transient initial improvement but ultimately experienced relentless disease progression, and one patient died. The overall prognosis was poor: one death, one vegetative state, and progressive disease in the remaining 5 patients.

**Conclusion:**

ROHHAD syndrome presents with highly heterogeneous clinical manifestations. Neuropsychiatric abnormalities may occur in parallel with or closely following rapid-onset obesity. Unexplained obesity and neuropsychiatric abnormalities in young children should raise high suspicion for this syndrome. Such patients warrant routine multidisciplinary monitoring. In the three patients who received rituximab, clinical responses were variable and transient, with no sustained long-term improvement observed. However, due to the small sample size and heterogeneous treatment protocols, no definitive conclusion regarding rituximab efficacy can be drawn.

## 1 Introduction

Rapid-onset Obesity with Hypothalamic dysfunction, Hypoventilation, and Autonomic Dysregulation (ROHHAD) syndrome is a rare pediatric neurological disorder with a poor prognosis. Approximately half of the patients have coexisting neural crest-derived tumors (ROHHAD-NET) (1). Diagnosis is relied on a constellation of characteristic clinical features. Due to its insidious early symptoms and highly heterogeneous disease course, ROHHAD syndrome is prone to misdiagnosis or delayed diagnosis in clinical practice.

In recent years, increasing attention has been paid to evaluating the clinical symptom spectrum and its evolution patterns. Wang et al. (2) reported 16 Chinese patients in 2024, demonstrating that autonomic dysregulation occurred in 100% of cases with diverse manifestations, suggesting its core diagnostic value. Regarding treatment, based on the hypothesized autoimmune etiology (3), immunomodulatory therapies such as rituximab have been attempted. Although isolated case reports have suggested short-term or partial efficacy of rituximab (4, 5), its precise efficacy and impact on long-term prognosis remain unclear. This study systematically reviews the clinical data of 7 children with ROHHAD syndrome. We aim to precisely characterize their clinical phenotypes and the chronological sequence of symptom evolution, with a particular focus on analyzing differential responses to rituximab therapy, thereby providing clinical evidence to facilitate early diagnosis and individualized treatment decisions.

## 2 Materials and methods

### 2.1 Study population

We retrospectively identified 7 children diagnosed with ROHHAD syndrome who received care in the pediatric neurology departments of two hospitals between 2010 and 2025. Diagnostic criteria were based on the major/minor criteria system used by Fava et al. (6). Definite ROHHAD: 3 major criteria, or 2 major + 2 minor criteria. Probable ROHHAD: 1 major + 4 minor criteria, or 2 major + 1 minor criteria. Major criteria include: (1) rapid-onset obesity; (2) central hypoventilation; (3) neural crest tumor. Minor criteria include: autonomic dysregulation, endocrinopathies (hyperprolactinemia, central hypothyroidism, adrenal insufficiency, etc.), water-balance abnormalities, strabismus, and behavioral disorders. Due to the retrospective study design, genetic testing was not available for all patients. However, the following alternative diagnoses were excluded based on clinical criteria: (1) PHOX2B-related congenital central hypoventilation syndrome—excluded by the absence of neonatal respiratory failure or ventilator dependence from birth; (2) Prader-Willi syndrome—excluded by the absence of neonatal hypotonia, feeding difficulties, and characteristic facial features; and (3) other structural or genetic causes of hypothalamic obesity—excluded by normal brain imaging in all patients and by the presence of multisystem autonomic dysfunction and neuropsychiatric abnormalities, features not characteristic of isolated hypothalamic obesity.

Brain imaging was performed in all patients to exclude structural lesions in the hypothalamic-pituitary region, with modalities varying across patients: Four patients underwent cranial MRI; one patient underwent cranial CT and hippocampal MRI at an outside hospital (both normal); two patients underwent pituitary MRI as part of endocrine evaluation (both normal, but hypothalamic regions not fully covered due to limited field of view). Detailed neuroimaging findings for each patient are summarized in Table 1. In addition, multi-phase contrast-enhanced CT of the chest, abdomen, and pelvis was performed before and after surgery to assess for neural crest tumors. This study protocol was approved by the Ethics Committee of our institution.

**Table 1:** Aggregated clinical characteristics of the cohort.

Feature	n (%)	Median age years (range)	Feature	n (%)	Median age years (range)
Demographics			Hypothalamic dysfunction		
Median age at onset	—	2.8 (2.0–7.9)	Hyperphagia	7/7 (100%)	3.1 (2.0–7.9)
Median age at diagnosis	—	3.8 (3.5–8.5)	Water–electrolyte imbalance	6/7 (85.7%)	3.5 (3.4–5.2)
Median diagnostic delay	—	1.2 (0.5–3.6)	Adrenal dysfunction	5/7 (71.4%)	3.6 (3.4–8.5)
Core features			Hyperprolactinemia	4/7 (57.1%)	3.6 (3.5–8.4)
Rapid-onset obesity	7/7 (100%)	3.2 (2.5–7.9)	Thyroid dysfunction	3/7 (42.9%)	3.4 (3.4–8.5)
Hypothalamic dysfunction	7/7 (100%)	3.1 (2.0–7.9)	Growth hormone axis abnormality	4/7 (57.1%)	4.0 (3.4–8.5)
Autonomic dysregulation	7/7 (100%)	3.4 (2.8–8.3)	Central precocious puberty	1/7 (14.3%)	4.0
Central hypoventilation	5/7 (71.4%)	3.5 (3.0–8.6)	Autonomic dysregulation		
Neural crest-derived tumors	4/7 (57.1%)	3.4 (3.3–8.4)	Temperature dysregulation	6/7 (85.7%)	3.4 (3.0–8.4)
Neuropsychiatric abnormalities	7/7 (100%)	3.1 (2.0–8.1)	Cardiovascular abnormality	5/7 (71.4%)	3.6 (3.2–8.6)
Other features			Abnormal sweating	4/7 (57.1%)	3.6 (3.5–8.4)
Cardiac involvement	4/7 (57.1%)	—	Gastrointestinal dysmotility	3/7 (42.9%)	3.6 (2.9–8.4)
Epileptic seizures	2/7 (28.6%)	—	Enuresis	2/7 (28.6%)	3.0 (2.9–3.5)
Elevated IL-6 (tested in 4)	2/4 (50%)	—	Pain insensitivity	1/7 (14.3%)	3.5

Data are presented as *n* (%) unless otherwise indicated.

### 2.2 Clinical data collection

Alveolar hypoventilation was assessed clinically through nocturnal polysomnography, pulse oximetry, or overnight blood gas monitoring. Nocturnal sleep hypoventilation was defined by any of the following criteria on overnight polysomnography: (1) apnea-hypopnea index > 5 events/hour; (2) partial pressure of carbon dioxide (PaCO₂) > 45 mmHg; or (3) oxygen saturation decreased by ≥ 4% from baseline levels. Oxygen desaturation alone was not considered equivalent to central hypoventilation.

A standardized clinical data collection form was developed to gather information including: (1) baseline demographic data; (2) age at first appearance and specific manifestations of each core symptom/sign; (3) timing and regimen details of immunotherapies, tumor resections, and other treatments; and (4) outcomes at last follow-up. Descriptive statistical methods were used for data analysis. Categorical data were presented as frequencies (n) and percentages (%), while continuous variables such as age of onset were described as medians (range). Diagnostic delay was calculated for each patient as the difference between age at diagnosis and age at symptom onset; the median of these individual delays was then reported.

## 3 Results

### 3.1 Baseline characteristics and diagnostic delay

Clinical data of 7 patients were listed in Table 1 and Figure 1. According to this classification, 6 patients met definite ROHHAD. One patient presented with 1 major criterion (obesity) and 2 minor criteria (autonomic dysregulation and behavioral disorder), which does not reach the threshold for probable ROHHAD; this case is presented as an incomplete/atypical phenotype.

**Figure 1:**
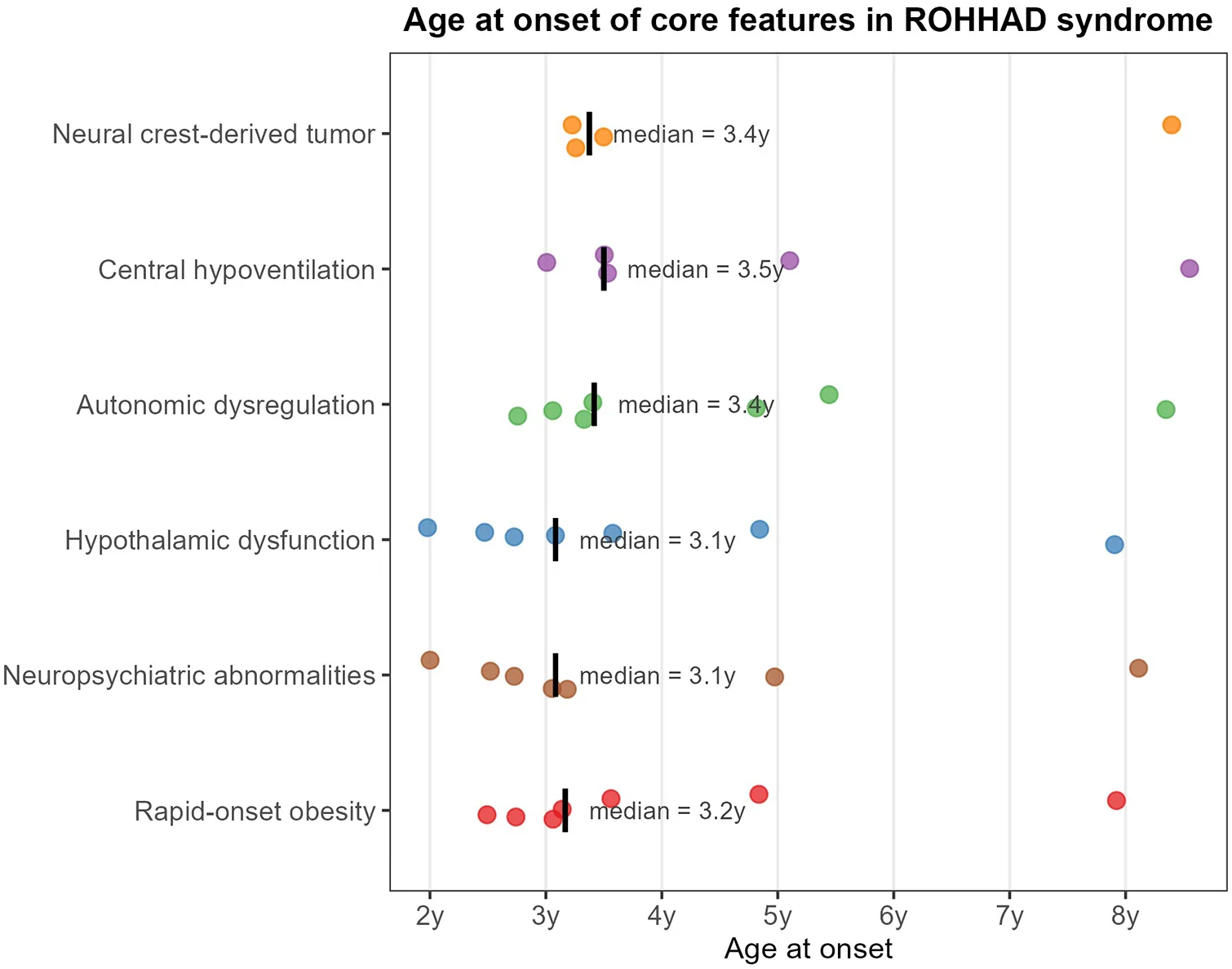
Age at onset of core features in ROHHAD syndrome. Points represent the age at onset of each symptom, with the same color indicating the same symptom. Short horizontal lines indicate the median age at onset for each symptom. Age is shown in years.

Among the 7 patients, there were 3 males and 4 females. The median age of onset was 2.8 years (2.0–7.9 years), the median age at initial presentation was 3.2 years (range 2.0–8.1 years), and the median age at diagnosis was 3.8 years (range 3.5–8.5 years). Individual diagnostic delays ranged from 6 to 43 months, with a median of 14 months. All patients (100%) exhibited the triad of rapid-onset obesity, hypothalamic dysfunction, and autonomic dysregulation.

Analysis of initial symptoms revealed that obesity/hyperphagia was the most common presenting manifestation (5 cases, 71.4%), followed by neuropsychiatric abnormalities (4 cases, 57.1%). These two could occur simultaneously or sequentially. Reasons for initial presentation were highly heterogeneous, mainly including unexplained fever (4 cases, 57.1%), neuropsychiatric abnormalities (2 cases, 28.6%), seizures (1 case, 14.3%), and lethargy/fatigue (1 case, 14.3%). Notably, none of the patients presented with obesity as the chief complaint at the initial visit, highlighting that this core sign is easily overlooked early on. Even at diagnosis, the chief complaints remained predominantly non-specific.

### 3.2 Core clinical features

#### 3.2.1 Rapid-onset obesity

All 7 patients exhibited rapid-onset obesity (significant weight gain within 6–12 months), with a median age of onset of 3.2 years (range 2.5–7.9 years).

#### 3.2.2 Hypothalamic dysfunction

All had hypothalamic dysfunction, with a median age of onset of 3.1 years (range 2.0–7.9 years). Hypothalamic dysfunction manifested across multiple endocrine axes. Hyperphagia was universal 7/7, 100%, though one patient exhibited late-stage appetite loss. The most frequent abnormalities involved water-electrolyte balance 6/7, 85.7%, adrenal axis 5/7, 71.4%, and hyperprolactinemia 4/7, 57.1%. Growth hormone axis abnormalities were observed in 4/7 patients (57.1%). Less common findings included thyroid dysfunction (3/7) and central precocious puberty (1/7).

#### 3.2.3 Autonomic dysregulation

was also universal, with a median onset of 3.4 years (range, 2.8–8.3 years). The most common feature was thermoregulatory impairment (6/7, 85.7%), including one patient with a peak fever of 42℃. Cardiovascular involvement (5/7, 71.4%) ranged from sinus tachycardia (>220 bpm) to paroxysmal hypertension. Other manifestations included abnormal sweating (4/7), gastrointestinal dysmotility (3/7), enuresis (2/7), and pain insensitivity (1/7).

#### 3.2.4 Central hypoventilation and respiratory manifestations

Five patients (71.4%) developed central hypoventilation, with a median onset age of 3.5 years (range 3.0–8.6 years). Respiratory support was required in 5 patients (71.4%). One patient was the most severe, with polysomnography-confirmed alveolar hypoventilation that progressed to respiratory failure with excessive airway secretions, requiring both non-invasive and invasive ventilatory support. Thus, respiratory failure with hypersecretion was observed in 1 of 7 patients (14.3%). One patient required non-invasive ventilation in BiPAP mode; however, this patient also had severe cardiomyopathy with pulmonary edema (EF 31%), and the need for respiratory support was likely multifactorial, related to both central hypoventilation and cardiac dysfunction. Three patients received oxygen therapy (nasal cannula or nocturnal oxygen). Two patient did not require respiratory support.

### 3.3 Key associated features

#### 3.3.1 Neural crest-derived tumors

Four patients (57.1%) had neural crest-derived tumors, with histopathological types including ganglioneuroblastoma and ganglioneuroma. The median age at tumor diagnosis was 3.4 years (range 3.3–8.4 years).

#### 3.3.2 Neuropsychiatric abnormalities

In this study, neurological abnormalities were defined as objective findings on neurological examination, electroencephalography (EEG), or neuroimaging (e.g., seizures, white matter lesions, movement disorders, cognitive decline on formal testing). Neuropsychiatric abnormalities were defined as behavioral, emotional, or psychiatric manifestations (e.g., irritability, aggression, social withdrawal, hallucinations, language regression, mood disturbances). Where both were present, they were classified separately and are reported in distinct categories. All 7 patients (100%) developed neuropsychiatric abnormalities, with a median age of onset of 3.1 years (range 2.0–8.1 years). Manifestations were diverse, including emotional irritability, aggression, social withdrawal, language regression, slowed reaction, lethargy, visual hallucinations, and regressive behavior.

### 3.4 Involvement of other systems

#### 3.4.1 Cardiac involvement

Four patients (57.1%) had cardiac abnormalities. One patient presented with mildly reduced left ventricular systolic function [ejection fraction (EF) 49%] and massive pericardial effusion. Another developed hypertrophic cardiomyopathy and heart failure (EF 33%–43%). A third had cardiomyopathy with severely reduced cardiac function (EF 31%) and pulmonary edema. The remaining patient developed heart failure in a later disease stage (EF 31%).

#### 3.4.2 Neurological manifestations

All 7 patients (100%) had neurological abnormalities. Two patients had definite epileptic seizures; one presented with generalized seizures and epileptic spasms, and the other with generalized tonic-clonic seizures. Neuroimaging findings in this cohort were heterogeneous. Four patients underwent cranial MRI, which revealed white matter lesions with bilateral basal ganglia abnormal signals in one patient, brain atrophy with features suggestive of reversible posterior leukoencephalopathy syndrome in another, bilateral periventricular and posterior horn T2 hyperintensities suggestive of unmyelinated white matter in a third, and no abnormalities in the remaining patient. One patient underwent cranial CT and hippocampal MRI at an outside hospital, both normal. Two patients underwent pituitary MRI, both normal. PET/CT was performed in three patients, all of whom demonstrated diffuse cerebral hypometabolism, with one patient also showing symmetrically increased metabolic activity in the basal ganglia. EEG was recorded in five patients. Among these, three patients exhibited slowing or loss of background rhythm, three patients showed increased slow waves, and three patients had epileptiform discharges (spike-slow waves and sharp-slow waves). In addition, one patient presented with movement disorders and language regression. These findings indicate a broad spectrum of neurological injury in this disease; imaging and electrophysiological studies are helpful for comprehensive assessment.

### 3.5 Inflammatory markers

Among the 7 patients, blood IL-6 levels were measured in 4. Of these, two patients had elevated levels (25 pg/mL and up to 100 pg/mL, respectively; normal <6.4 pg/mL). The other two patients had normal serum and cerebrospinal fluid IL-6 or no abnormalities on blood cytokine testing. This suggests that inflammatory activation is present in some but not all patients.

### 3.6 Treatment response and outcomes

The treatments of the 7 patients are listed in Table 2. First-line immunotherapy response:Six patients (85.7%) received first-line immunosuppressive therapy early in the disease course, including high-dose corticosteroid pulses and/or intravenous immunoglobulin, but the efficacy was either not durable or only partial and transient, suggesting that single or traditional immunosuppressive regimens are insufficient to control disease progression.

**Table 2:** Treatment and outcomes of the cohort.

Treatment category	Details	n (%)
First-line immunotherapy	Corticosteroids and/or IVIG	6/7 (85.7%)
Corticosteroids	Methylprednisolone pulse (15 mg/kg/d–400 mg/d × 2–3 courses), followed by oral prednisone (1.2–1.75 mg/kg/d) 3–6 months	5/7 (71.4%)
IVIG	2 g/kg, given as 1–2 episodes in some patients	4/7 (57.1%)
Second-line immunotherapy	Rituximab	3/7 (42.9%)
Rituximab	1 case: 375 mg/m² weekly × 4 doses; 1 case: 375 mg/m² single dose; 1 case: 750 mg/m² single dose	3/7 (42.9%)
Tumor treatment	Surgical resection, with or without postoperative chemo-radiotherapy	4/7 (57.1%)
Surgical resection	All patients with neural crest-derived tumors	4/7 (57.1%)
Postoperative chemo-radiotherapy	—	1/7 (14.3%)
Respiratory support	Non-invasive or invasive ventilation, or oxygen therapy	5/7 (71.4%)
Non-invasive ventilation	BiPAP or non-invasive ventilation	1/7 (28.6%)
Invasive ventilation	—	1/7 (14.3%)
Oxygen therapy	Nasal cannula or nocturnal oxygen	3/7 (57.1%)
Other treatments		
Topiramate	Appetite suppression	1/7 (14.3%)
Desmopressin	Diabetes insipidus	1/7 (14.3%)
Antihypertensives	—	2/7 (28.6%)
Antiepileptic drugs	Perampanel or clonazepam	2/7 (28.6%)
Antipsychotics	Haloperidol, aripiprazole, olanzapine, or bromocriptine	4/7 (57.1%)
Diuretics/inotropes	Milrinone, diuretics	1/7 (14.3%)
Outcomes		
Death	—	1/7 (14.3%)
Vegetative state	—	1/7 (14.3%)
Persistent disease progression	—	5/7 (71.4%)
Lost to follow-up	—	3/7 (42.9%)
Alive at last follow-up	—	3/7 (42.9%)

Data are presented as *n* (%).

Response to tumor resection: All 4 patients with tumors underwent tumor resection. In one patient, the interval between fever episodes lengthened and neuropsychiatric abnormalities diminished postoperatively, but the disease ultimately progressed and the patient died. The remaining 3 patients showed no relief of neurological symptoms and continued to experience disease progression.

#### 3.6.1 Response to rituximab therapy

Three patients received rituximab. One patient received rituximab at a disease duration of approximately 15 months, approximately 6 months after tumor resection, at a dose of 375 mg/m² weekly for 4 doses. Transient improvement in behavioral abnormalities and fever intervals was observed during the first 3 months, but disease progression resumed thereafter. The patient died during follow-up due to cardiopulmonary arrest. CD19/CD20 monitoring was not performed. Another patient received rituximab in combination with corticosteroids at a disease duration of approximately 18 months, approximately 2 months after tumor resection, as a single dose of 750 mg/m². Emotional lability and nocturnal oxygen desaturation frequency improved transiently, and excessive weight gain appeared partially contained during the first 6 months, with no weight gain observed during the first 3–4 months post-treatment. However, at 12-month follow-up, neuropsychiatric abnormalities and seizures persisted with no cognitive progress. CD19/CD20 levels were not systematically monitored. A third patient received rituximab at a disease duration of approximately 7 months, approximately 1 month after retroperitoneal tumor resection, as a single dose of 375 mg/m². Mood showed mild transient improvement, but disease progression continued. At last follow-up, the patient was alive with poor cognition. CD19/CD20 monitoring was not performed. (Table 2)

#### 3.6.2 Other medications

One patient received add-on topiramate, which slightly controlled appetite but with limited effect.

#### 3.6.3 Overall prognosis

The overall prognosis of this patient cohort was poor: one death, one vegetative state, and the remaining 5 patients with persistent disease progression.

## 4 Discussion

Through a systematic analysis of 7 patients with ROHHAD syndrome, this study deepens the understanding of the natural history and heterogeneity of this disease from four dimensions: clinical manifestations, temporal evolution, multisystem involvement, and immunotherapy responses.

### 4.1 Core clinical features and diagnostic implications

The clinical features of our cohort confirm that autonomic dysregulation is an indispensable core component of this syndrome (2). Its incidence was 100%, and all patients had more than two different types of symptoms, consistent with previous reports. Notably, neuropsychiatric abnormalities represent an early symptom of this disease.

Regarding the order of symptom appearance, the classic view holds that the typical evolution of ROHHAD syndrome is: obesity→autonomic dysregulation→hypothalamic dysfunction→hypoventilation (1). However, Wang et al. (2) systematically analyzed 16 cases and revised this understanding, proposing a more complete sequence: obesity→neuropsychiatric abnormalities→autonomic dysregulation→hypothalamic dysfunction→ tumor→hypoventilation. The median age of onset for neuropsychiatric abnormalities was 3.21 years, second only to obesity (3.17 years), and in some patients, behavioral abnormalities could precede obesity by up to 2 years.

In our cohort, the median age of onset of neuropsychiatric abnormalities (3.1 years) was almost concurrent with that of rapid-onset obesity (3.2 years), rather than strictly following the “obesity first” sequence. In one patient, behavioral abnormalities and obesity appeared simultaneously in early childhood. In another, the first disease episode presented with motor and language impairment and emotional abnormalities, while obesity did not appear until the second episode. These findings suggest that neuropsychiatric abnormalities and rapid-onset obesity can occur in parallel or closely sequentially, and that some patients may even present with behavioral abnormalities as the first manifestation.

These observations provide important clues for early diagnostic strategies: in young children with unexplained neuropsychiatric symptoms accompanied by significant hyperphagia or weight gain, the possibility of ROHHAD syndrome should be highly suspected. Notably, none of the patients in this cohort presented with obesity as the chief complaint at initial presentation, suggesting that clinicians’ early recognition of and attention to obesity as a core sign needs improvement. Diagnostic delay was substantial in this cohort, with a median of 14 months (range, 6–43 months), further highlighting the urgency of increasing clinical vigilance.

Furthermore, this study expands the understanding of the hypothalamic dysfunction spectrum. In addition to hyperphagia, water-electrolyte imbalance (85.7%), adrenal dysfunction (71.4%), and hyperprolactinemia (57.1%) were very common, suggesting that comprehensive pituitary-target gland axis evaluation should be performed in confirmed patients. Consistent with our findings, a nationwide observational cohort study from Japan also reported significantly elevated serum prolactin levels at initial consultation in all cases, suggesting that hyperprolactinemia may be an important clue for early diagnosis (7).

### 4.2 Multisystem involvement and clinical management priorities

ROHHAD syndrome often involves multiple systems. Our data demonstrate that cardiac, neurological, and neuropsychiatric abnormalities are particularly prominent and are key factors affecting prognosis and quality of life. The incidence of cardiac involvement was 57.1% (4/7), which can manifest as cardiomyopathy, heart failure, pericardial effusion, and pulmonary edema. Two patients developed severe heart failure (EF 31%), suggesting that cardiac complications are an important factor leading to poor prognosis. These cardiac abnormalities probably be interpreted as secondary consequences of severe autonomic dysregulation and respiratory dysfunction, rather than primary myocardial manifestations of ROHHAD. Therefore, routine cardiac ultrasound and cardiac function monitoring should be performed in ROHHAD patients, with dynamic follow-up during the disease course to enable early identification and intervention for cardiac dysfunction.

Neuropsychiatric abnormalities occurred in 100% (7/7), mainly manifesting as emotional irritability, aggression, social withdrawal, language regression, lethargy, and visual hallucinations. These abnormalities not only severely affect patients quality of life but also impose a significant burden on family caregivers. Clinical management should emphasize emotional and behavioral interventions, with collaboration with child psychiatrists when necessary.

Neurological abnormalities were present in all 7 patients, but epileptic seizures were seen in 2 (28.6%) patients. Most patients presented with cognitive decline, movement disorders, and diffuse brain injury on imaging. Therefore, even without clinical seizures, routine EEG and neuroimaging evaluation should be performed. For patients with seizures, antiepileptic treatment should be initiated promptly.

In summary, the management of ROHHAD syndrome requires a multidisciplinary approach involving cardiology, neurology, endocrinology, and psychiatry to address its complex systemic involvement.

### 4.3 Immune mechanisms and response to rituximab therapy

The etiology of ROHHAD syndrome has long remained unclear. In recent years, increasing evidence supports an autoimmune mechanism playing a central role in its pathogenesis (8, 9).

The subfornical organ (SFO), a sensory circumventricular organ lacking a blood-brain barrier, has become a research focus. Studies have detected anti-SFO antibodies in the serum of ROHHAD patients (10), suggesting that autoantibodies may attack this “brain window,” leading to hypothalamic-pituitary dysfunction. In 2022, Mandel-Brehm et al. (11) identified anti-ZSCAN1 antibodies in patient serum and CSF, and demonstrated ZSCAN1 antigen expression in neuroblastic tumor and brain tissue. Nakamura-Utsunomiya et al. (2024) (12) identified ZSCAN1 as a target antigen in ROHHAD patients without tumors, showing that over 85% of ROHHAD patients were anti-ZSCAN1 antibody positive by ELISA, with higher antibody titers in those with poor prognosis, suggesting that this antibody may serve as a diagnostic marker for ROHHAD syndrome independent of tumor. More critically, Serafim et al. (2024) (13) found that 83.3% of ROHHAD patients had detectable anti-ZSCAN1 antibodies only in cerebrospinal fluid but not in serum, suggesting that intrathecal autoantibody synthesis may be a potential disease mechanism. In a Japanese nationwide study of 30 patients clinically diagnosed with ROHHAD(-NET) syndrome, the positivity rate for serum anti-ZSCAN1 and anti-Nax antibodies was 70% (7). Notably, anti-ZSCAN1 antibody titers were significantly higher in patients with neural crest tumors than in those without (7). Additionally, some patients have anti-Nax antibodies (14), which may explain the common presentations of hypernatremia and reduced thirst sensation. These findings collectively suggest that immune responses targeting the SFO and its neural network may contribute to the pathogenesis of this disease. However, anti-ZSCAN1 antibodies should be interpreted as potential markers of an antigen-specific autoimmune response rather than direct causes of hypothalamic injury, as ZSCAN1 is an intracellular antigen. Notably, anti-ZSCAN1 antibodies were not measured in our 7 patients due to insufficient stored samples, which we acknowledge as a limitation.

However, autoantibodies are not the sole immune issue underlying this disease. Fava et al. (6) analyzed peripheral blood lymphocyte subsets from 11 ROHHAD patients and found significantly elevated total T lymphocyte counts (mainly CD4+ T cells), increased regulatory B cells (Breg) and type-1 regulatory T cells (Tr1), but no significant difference in conventional regulatory T cells (Treg). The increase in Breg and Tr1 cells may represent a compensatory response to ongoing immune activation rather than enhanced regulatory activity. In addition, elevated IL-8 and MCP-1 levels were observed, suggesting potential involvement of innate/myeloid inflammatory pathways, although serum elevations do not prove CNS infiltration. These findings suggest that ROHHAD may involve both adaptive and innate immune disturbances beyond humoral immunity alone.

Exploration of rituximab efficacy from an immunological perspective:Rituximab is a CD20-targeting B-cell depleting antibody that theoretically can eliminate autoantibody-producing B cells. Hawton et al. (2022) reported a ROHHAD patient who showed significant improvement after rituximab treatment (4), providing preliminary evidence for its clinical use.

However, in our cohort, all 3 patients who received rituximab showed unsatisfactory long-term outcomes, with 2 deteriorating in the long term and 1 death. Several factors may explain this apparent inefficacy. First, ROHHAD involves T-cell-mediated cellular immune abnormalities, and rituximab only depletes B cells without directly inhibiting T-cell activation or effector function (6). Second, the presence of intrathecal autoantibody synthesis suggests that the central nervous system itself is a target of immune attack, and rituximab, as a large molecule antibody, has difficulty effectively crossing the blood-brain barrier. Third, rituximab does not eliminate CD20-negative long-lived plasma cells, which may sustain autoantibody production despite peripheral B-cell depletion. In addition, downstream inflammatory pathways or irreversible hypothalamic and brainstem neuronal injury may contribute to continued disease progression despite suppression of the initial adaptive immune response.

An important distinction should also be made between immunological response and clinical recovery. Suppression of immune activity may not translate into functional recovery if hypothalamic, brainstem, or autonomic circuits have already been irreversibly damaged. Behavioral abnormalities or hyperphagia may respond differently from central hypoventilation or autonomic dysfunction. Therefore, poor long-term neurological outcome should not be interpreted as proof that rituximab had no immunological effect, especially without serial biomarker monitoring. This distinction helps explain the apparent transient improvements observed in our cohort.

### 4.4 Limitations of tumor resection and the immune initiation hypothesis

In our cohort, the 4 patients with tumors showed no relief of neurological symptoms after tumor resection and continued to progress. This phenomenon is consistent with the hypothesis that tumors act as autoimmune triggers: antigens expressed by tumor cells (e.g., ZSCAN1) may break immune tolerance and initiate an autoimmune response; once the immune process is activated, the response can be self-sustaining even after tumor resection (15). Furthermore, tumor resection in four patients failed to halt disease progression, consistent with the hypothesis that the autoimmune cascade becomes self-sustaining and independent of the primary tumor once triggered.

### 4.5 Limitations

This study is subject to inherent limitations, given its retrospective, two-center design and the small sample size. However, it is precisely the significant heterogeneity within this small sample that provides critical clinical insights: in the absence of predictive biomarkers, immunotherapy should be regarded as exploratory individualized treatment, and expectations for efficacy should be tempered.

## 5 Conclusion

The clinical phenotypes of ROHHAD patients in this cohort are consistent with literature reports. Autonomic dysregulation is common and diverse, but more importantly, neuropsychiatric abnormalities may occur in parallel with or closely following rapid-onset obesity, warranting clinical attention. Multisystem involvement (cardiac, neurological, neuropsychiatric) is prominent, necessitating multidisciplinary, individualized management strategies. Immunological mechanistic studies have revealed the important roles of anti-ZSCAN1 antibodies and T-cell abnormalities in pathogenesis. The long-term outcomes of rituximab therapy in this cohort were unsatisfactory in the three treated patients. However, given the small sample size and heterogeneous treatment conditions, the efficacy of rituximab requires confirmation in larger cohorts with standardized protocols. Future research should focus on exploring immune subtype-guided individualized treatment regimens.

## Data Availability

The raw data supporting the conclusions of this article will be made available by the authors, without undue reservation.
